# Novel hyaluronic acid oligosaccharide-loaded and CD44v6-targeting oxaliplatin nanoparticles for the treatment of colorectal cancer

**DOI:** 10.1080/10717544.2021.1914777

**Published:** 2021-05-11

**Authors:** Wenlong Du, Xiaoping Yang, Shenfu He, Jia Wang, Yuanxian Guo, Bangguo Kou, Yongjie Jiang, Pan Bian, Bingtai Li, Lanning Yin

**Affiliations:** a Department of General Surgery, The Second Hospital of Lanzhou University, Lanzhou, PR China; b Department of General Surgery, Xigu People’s Hospital, Lanzhou, PR China

**Keywords:** Hyaluronic acid oligosaccharide, CD44v6, oxaliplatin, drug delivery system, colorectal cancer

## Abstract

Oxaliplatin resistance is one of the main causes of failed colorectal cancer treatment, followed by recurrence and metastasis. In this study, we found that colorectal cancer cells secrete a high level of hyaluronic acid (HA), which interacts with its receptor CD44v6 to mediate colorectal cancer resistance to chemotherapy. HA oligosaccharide (oHA) is a degradation product of HA. We found that it competitively binds to CD44v6, reversing the HA–CD44v6-mediated effect of HA on oxaliplatin resistance. In addition, oHA showed no toxicity or immunogenicity but exhibited good biocompatibility and tumor-targeting capability. Therefore, we synthesized oHA-loaded oxaliplatin liposome nanoparticles (oHA-Lipid-Oxa) using a thin-film hydration method. The cytotoxicity of oHA-Lipid-Oxa was assessed in vitro using flow cytometry, which revealed greater lethality than oxaliplatin alone. Finally, we established a tumor-bearing nude mouse model and separately injected oHA-Lipid-Oxa, Lipid-Oxa, Oxa, oHA, and phosphate-buffered saline (PBS) into the tail vein to observe the antitumor effects of nanoparticles in vivo. The oHA-Lipid-Oxa group exhibited the highest tumor suppression rate, but the weight loss was not obvious. Hematoxylin and eosin staining showed greatest lymphocyte and macrophage infiltration in the oHA-Lipid-Oxa group. Moreover, oHA-Lipid-Oxa induced tumor cell apoptosis and necrosis most robustly compared with the other groups. We showed that oHA-Lipid-Oxa has excellent histocompatibility and CD44v6-targeting capabilities, thus greatly increasing the sensitivity to oxaliplatin and reducing adverse reactions. Accordingly, oHA-Lipid-Oxa has a broad potential for therapeutic application.

## 1. Introduction

Colorectal cancer is the third most common malignant tumor. There were approximately 15,000 new cases and more than 50,000 deaths from this disease in the United States in 2019 (Siegel et al., 2019). Although the treatment of colorectal cancer has greatly improved over time, it remains one of the leading causes of cancer-related deaths. Oxaliplatin-based chemotherapy is the first-line treatment for colorectal cancer (Gupta et al., 2019). Oxaliplatin is a third-generation platinum chemotherapeutic drug that can form Pt-DNA adducts with DNA chains to produce inter-chain cross-linking and intra-chain cross-linking, resulting in DNA damage (Fong 2016; Yuan et al., 2020). It can also inhibit the synthesis of DNA and RNA, and trigger systemic immunoreactions, leading to apoptosis (Perego and Robert 2016). However, the prognosis of patients with colorectal cancer is often less optimistic than expected, mainly because of oxaliplatin resistance (Kopetz et al., 2010). In addition, chemotherapy resistance is a key prognostic factor for colorectal cancer, in that approximately 40%–50% of patients receiving chemotherapy for stages II and III colorectal cancer eventually develop chemotherapy resistance and disease recurrence (de Mestier et al., 2014). Therefore, the improvement of oxaliplatin resistance is essential for the treatment of colorectal cancer.

Usually, oxaliplatin resistance is caused by ‘pump’ and ‘non-pump’ mechanisms. Pump mechanisms are triggered mainly by ATP-binding proteins, including P-gp1, breast cancer resistance protein, and multidrug resistance-related protein (Wang et al., 2015; Lu et al., 2017). This transmembrane transport protein can expel chemotherapeutic drugs from cells, thus reducing the intracellular drug concentration. Non-pump resistance mechanisms mainly include (1) overexpression of DNA repair systems, including mismatch repair and nucleotide excision repair (Kirschner and Melton 2010; Slyskova et al., 2018), which quickly repair damaged DNA during the G2/M phase of the cell cycle, thus producing drug resistance (Cohen et al., 2015); (2) inhibition of apoptosis, including upregulation of anti-apoptotic proteins such as Bcl2, NF-κB, and p53 (Ruiz de Porras et al., 2016; Smith and Macleod 2019); (3) detoxification via glutathione-S transferase and cytochrome P450 (Noda et al., 2012; Lin et al., 2016); and (4) multidrug resistance mediated by cancer stem cells, epithelial–mesenchymal transition, and altered tumor microenvironment (Zhang et al., 2015; Steinbichler et al., 2018; Chen et al., 2019).

Hyaluronic acid (HA) is a linear polysaccharide composed of D-glucuronic acid and N-acetylglucosamine linked by β-1,3-glycosidic and β-1,4-glycosidic bonds with molecular weights of 10^5^–10^7^ Da (Wang et al., 2011). HA reportedly can interact with CD44v6, one of the important markers of colorectal cancer stem cells, which are involved in intracellular signal transduction pathways that promote cancer cell survival and proliferation, as well as chemoresistance (Ni et al., 2014; Cyphert et al., 2015; Song et al., 2019). HA oligosaccharide (Ghosn et al., 2019) is a degradation product of HA that can competitively bind to CD44v6, thereby reversing chemotherapy resistance, and oHA serves as a good carrier for controlled drug delivery(Cui et al., 2009; Zhao et al., 2014).

In this study, we aimed to design novel oHA-loaded and CD44v6-targeting oxaliplatin nanoparticles (oHA-Lipid-Oxa) for the treatment of colorectal cancer. On the one hand, oHA-Lipid-Oxa can target CD44v6, which can improve the accuracy of colorectal cancer chemotherapy. On the other hand, encapsulating oxaliplatin in liposomes can reduce toxic and side effects, improve enhanced permeability and retention effect (EPR), and boost the efficiency of chemotherapy. First, we assessed the expression levels of HA and CD44v6 in colorectal cancer cell lines to determine their involvement in oxaliplatin chemoresistance and whether oHA can competitively replace HA. Second, we constructed and characterized oHA-Lipid-Oxa. Finally, we evaluated the therapeutic effects of oHA-Lipid-Oxa by flow cytometry and in vivo experiments.

## 2. Materials and methods

### 2.1. Materials

Fetal bovine serum and pancreatin were purchased from Gibco. RIPA lysis buffer was purchased from Solabel Technology Co., Ltd. (Beijing, China). BCA protein assay and Tris-Tricine-SDS-PAGE loading buffer were purchased from Biyuntian Biotechnology Co., Ltd. (Shanghai, China). A MTT assay kit was purchased from BioFroxx (Shanghai, China). The annexin V-FITC/propidium iodide double staining apoptosis detection kit was purchased from Kaiji Biotechnology Co., Ltd. (Nanjing, China). CD44v6 antibody was purchased from Abcam. oHA-4 (a hyaluronic acid including four saccharide residues) was purchased from Creativepegworks. 4-methylumbelliferone (4-MU) was obtained from MedChemExpress. BS3 was purchased from Thermo Fisher Scientific. A human hyaluronic acid enzyme-linked immunosorbent assay(ELISA) kit was purchased from Jiancheng Co., Ltd. (Nanjing, China). Oxaliplatin was obtained from Meilun Biotechnology Co., Ltd. (Dalian, China). 1,2-Dilauroyl-sn-Glycero-3-Phosphoethanolamine (DLPE) and 1,2-Dilauroyl-sn-Glycero-3-Glycerol (DLPG) were purchased from Avanti (Alabaster, AL, USA). BALB/c male nude mice were purchased from Sbefu Biotechnology Co., Ltd. (Beijing, China).

### 2.2. Cell culture

Colorectal cancer cell lines (RKO and HT29) and a normal colon epithelial cell line (NCM460) were purchased from the Shanghai Cell Bank of Chinese Academy of Sciences. All cells were cultured in DMEM (pH 7.2) at 37 °C in a humidified atmosphere containing 5% CO_2_. The medium was supplemented with 10% (v/v) fetal bovine serum, 100 U/mL penicillin, and 100 µg/mL streptomycin.

### 2.3. Detection of CD44v6 expression

The expression of CD44v6 in each cell line was detected by flow cytometry. The cells were digested with EDTA and centrifuged. The supernatant was discarded, and the cells were washed twice in phosphate-buffered saline (PBS). Cell counts were recorded and the cell densities adjusted. All cells were seeded at 10^6^/well and incubated with the CD44v6 antibody according to the manufacturer’s instructions. The control group was incubated with a similar volume of control antibody. The cells were incubated in the dark at room temperature for 30 minutes, washed twice with PBS, and resuspended in 500 μL PBS for detection by flow cytometry.

### 2.4. ELISA detection of HA expression

The expression of HA in each cell line was examined by ELISA. Wells of a 96-well plate were designated as blanks, standards, and samples. Blank wells contained 50 μL standard diluent, and the remaining wells contained 50 μL standard solution or sample. Detection of the antibody signal was performed according to the ELISA kit instructions, and the optical density (OD) at 450 nm of each well was recorded.

### 2.5. Cytotoxicity assay

To measure the toxicity of oxaliplatin in colorectal cancer cells, RKO and HT-29 cells at the logarithmic growth stage were seeded in 96-well plates at 5 × 10^3^/well. Blank wells were also established, and all cells were cultured overnight at 37 °C. The cells were then treated with PBS or 1, 2.5, 10, 50, or 100 μM oxaliplatin. After 24 h, 10 μL MTT was added to each well, and the OD at 568 nm of each well was recorded.

### 2.6. Western blot and flow cytometry to detect oHA competitive substitution

The competitive substitution effect of oHA on HA was detected by western blot and flow cytometry. First, the protein concentrations of the samples were determined by bicinchoninic acid assay, and equivalent protein amounts (approximately 40 μg) were separated by electrophoresis. Proteins were transferred to polyvinylidene difluoride membranes and blocked with a skim milk solution for 1 h. The membranes were then incubated overnight at 4 °C with a primary antibody against CD44v6. Subsequently, membranes were washed with Tris-buffered saline containing 0.1% Tween-20, incubated with horseradish peroxidase-conjugated antibody for 1 h, and developed using an enhanced chemiluminescence kit.

RKO cells in good growth conditions were seeded in 6-well plates at 2.5 × 10^5^/well and cultured overnight at 37 °C. The cells were then treated with oxaliplatin only, oxaliplatin + 62.5 μg/mL oHA, oxaliplatin + 125 μg/mL oHA, or oxaliplatin + 250 μg/mL oHA. After 24 h, apoptosis was detected by flow cytometry.

### 2.7. Preparation of Lipid-Oxa

Lipid-Oxa was prepared as reported previously. First, 18 mg DLPE and 2 mg DLPG were added to 3 mL dichloromethane to achieve dissolution in oil phase. Second, 1 mL oxaliplatin solution (2.5 mg/mL) was gradually added to the oil phase and mixed with a probe ultrasonic detector (150 W; 2 min) to prepare an oil-in-water emulsion. The emulsion was placed on a rotary evaporator at room temperature. After the emulsion had been rotated and mixed for 10 min, it was dried for 4 h under vacuum conditions until the organic solvent had been completely evaporated to form a lipid film. Finally, the film was dissolved in PBS (pH 7.4) by water bath ultrasonication and extruded through polycarbonate membranes with a 400, 200, or 100-nm pore size to obtain oxaliplatin nanoparticles. The Lipid-Oxa was stored at 4 °C after ultrafiltration to a predetermined volume.

### 2.8. Preparation of oHA-Lipid-Oxa

oHA-4 (10 mg) was dissolved in disodium phosphate buffer solution (pH = 5.5), mixed with 1-ethyl-3-(3-dimethylaminopropyl) carbodiimide (EDC), and incubated at room temperature for 2 h to form a carboxyl-activated oHA solution with a concentration of 2 mg/mL. Oxaliplatin liposome nanoparticles were obtained according to the above procedures and then fully dissolved in carboxyl-activated oHA solution by water bath ultrasonication. The pH of the solution was adjusted to 9 by the addition of NaOH. Then, the lipid film was extruded through polycarbonate membranes with a 400, 200, or 100-nm pore size and stored overnight at 4 °C. The next day, oHA-modified oxaliplatin liposome nanoparticles were obtained by dialysis with 1 L PBS (pH = 7.4) in a dialysis bag with a molecular weight cutoff of 10 kDa. oHA-Lipid-Oxa was concentrated by ultrafiltration to a predetermined volume and stored at 4 °C.

### 2.9. Characterization of Lipid-Oxa and oHA-Lipid-Oxa

The zeta potential, polydispersity index, particle size, and distribution of the two samples were detected using a Malvern laser particle size analyzer (UK). The morphologies of Lipid-Oxa and oHA-Lipid-Oxa were observed by transmission electron microscopy. Lipid-Oxa and oHA-Lipid-Oxa solution (0.1 mg/mL) were added to 5-mL centrifuge tubes and incubated at room temperature. The particle sizes were measured at 0, 2, 4, and 7 days, and the solution clarity was observed. The particle sizes and their potential differences in the samples were detected using the Malvern laser particle size analyzer to determine the stability of the oxaliplatin liposome nanoparticles in vitro.

### 2.10. Detection of drug loading and oHA in oxaliplatin liposomes

A standard solution was prepared by diluting oxaliplatin with ddH_2_O. The absorption peak area at 230 nm was determined by high-performance liquid chromatography (HPLC), and a standard curve was established. The encapsulation efficiency and drug loading of oxaliplatin liposomes were calculated using the following formulas: encapsulation efficiency = weight of oxaliplatin in the micelle/weight of the feeding oxaliplatin × 100%; and drug loading = weight of oxaliplatin in the micelle/weight of the micelle × 100%. oHA-4 was dissolved in ddH_2_O to prepare a series of standard solutions (0.05, 0.1, 0.2, 0.4, and 0.8 mg/mL), with the addition of borax sulfate solution and carbazole reagent. The absorbance at 490 nm of the solutions was measured and plotted against the oHA-4 concentration to generate a standard curve. Based on this standard curve, the amount of oHA-4 on the oxaliplatin liposome surface was calculated as the weight of oHA in the micelle/weight of the micelle × 100%.

### 2.11. Flow cytometry

RKO cells in the logarithmic growth stage were seeded at 2.5 × 10^5^/well and cultured overnight at 37 °C. Cells were treated with PBS, oHA, Oxa, Lipid-Oxa, or oHA-Lipid-Oxa, after which they were digested in 0.25% trypsin without EDTA and incubated with 5 μL annexin V-FITC and propidium iodide. Apoptosis was detected by flow cytometry.

### 2.12. Antitumor activity of oHA-Lipid-Oxa micelles *in vivo*

The experimental animal procedures were reviewed by the Second Hospital of Lanzhou University Animal Care and Use Committee. Male nude mice (4–6 weeks old) were adapted for 7 days, after which RKO cells cultured until the logarithmic growth stage were inoculated into the right flank. Experiments were performed once the tumor volume reached approximately 70 mm^3^. The mice were randomized into five treatment groups: PBS, oHA, Oxa, Lipid-Oxa, or oHA-lipid-Oxa (oxaliplatin concentration: 5 mg/kg). The mouse weight and tumor volume were recorded at 3-day intervals. Tumor volume was calculated as (a × b^2^)/2, where a is the largest diameter and b the smallest diameter. Nude mice were euthanized after five treatments, and the tumors were excised. The tumor tissue was fixed in 4% paraformaldehyde overnight and sliced into 5-mm-thick sections for hematoxylin and eosin staining. The histological staining was observed at 200× using an Olympus microscope (Japan).

### 2.13. Statistical analysis

All data are presented as the mean of three independent experiments. Comparisons between groups were performed using unpaired t-tests. A p value < 0.05 was considered statistically significant.

## 3. Results and discussion

### 3.1. Cd44v6 and HA expression in colorectal cancer cell lines

**Figure 1.**
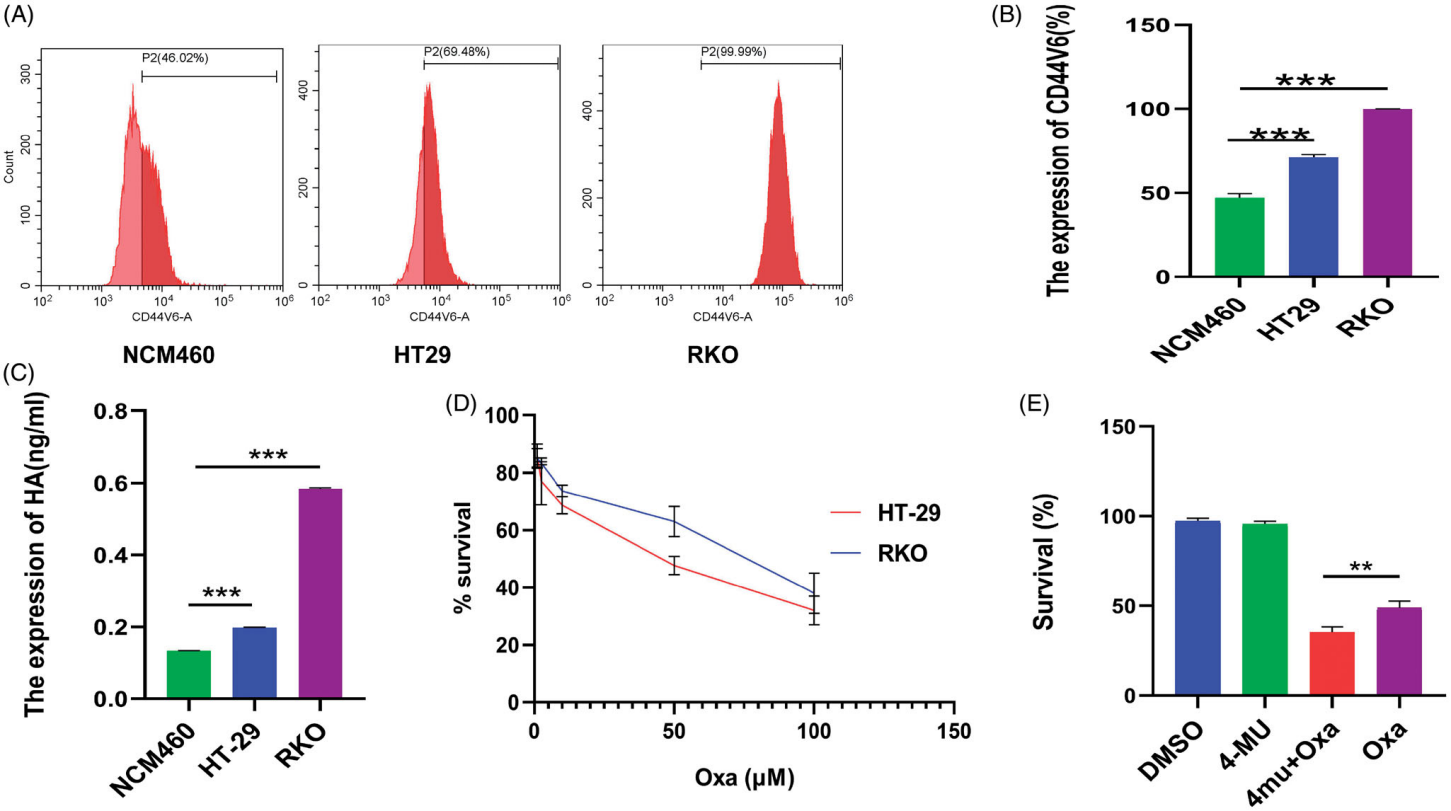
Interaction between HA and CD44v6 increased chemotherapy resistance in colorectal cancer cells. (A,B) Expression of CD44v6 in the indicated cells (***p < .001, compared with NCM460 cells). (C) Expression of HA in the indicated cells (***p < .001, compared with NCM460 cells). (D) Cytotoxicity of oxaliplatin in the indicated cells. RKO cells were more resistant to oxaliplatin than HT-29 cells. (E) RKO cells were treated with 0.05 mM 4MU or 0.05 mM 4MU + 10 μM Oxa for 72 h. DMSO was used as the vehicle control. The 4-MU can sensitize RKO cells to Oxa (**p < .01). All results are expressed as the mean ± SD of three independent experiments.

Chemotherapy resistance is a major cause of chemotherapy failure in colorectal cancer (Van der Jeught et al., 2018). Therefore, it is important to improve the curative effect of malignant tumor drugs by identifying and controlling the source of chemotherapy resistance. The mechanism of chemotherapy resistance is complex and has not been fully elucidated. Some malignant tumors secrete high levels of HA and exhibit chemotherapy resistance mediated by the CD44v6 receptor (Ma et al., 2019). In this study, we evaluated the expression levels of HA and CD44v6 in normal colon epithelial cells (NCM460) and colorectal cancer cells (HT-29 and RKO). The results showed that the expression levels of HA and CD44v6 were higher in the colorectal cancer cells than in normal colon epithelial cells (Figure 1(A–C)). The cytotoxicity of oxaliplatin was investigated in HT-29 and RKO cells by the MTT assay. The IC50 values of the two cell lines at 24 h were 40 µM and 74 µM, respectively. It can be found that higher expression of HA was associated with lower oxaliplatin sensitivity (Figure 1(D)). Therefore, we suspected that HA may reduce oxaliplatin sensitivity. Accordingly, RKO cells with high expression of HA and CD44v6 were divided into four groups and treated with dimethyl sulfoxide (DMSO), 0.05 mM 4-MU, 10 µM Oxa, or 0.05 mM 4-MU + 10 µM Oxa. The proliferation rates of the cells in these four treatment groups were 97 ± 1.23%, 96 ± 1.27%, 49 ± 2.93%, and 35 ± 2.41%, respectively. The 4-MU which is a HA synthase inhibitor can increase oxaliplatin sensitivity, suggesting that HA is involved in the resistance of RKO cells to oxaliplatin (Figure 1(E)). Yang et al. reported that breast cancer cells produce high levels of HA, which forms a ‘sugar coat’ barrier mediated by the CD44v6 receptor. When chemotherapy drugs approach tumor cells, drug resistance occurs because of the ‘sugar coat’ blocking effect (Yang et al., 2013). In addition, HA–CD44v6 interacts with protein kinase C to promote the expression of the cancer stem cell markers Nanog and microRNA21, which downregulate tumor suppressor factors and upregulate the expression of apoptosis inhibitor proteins and multidrug resistance-related protein (Bourguignon et al., 2009). HA also enhances the CXCL12-induced CXCR4 signaling pathway and promotes both vascular budding and angiogenesis (Fuchs et al., 2013).

### 3.2. Competitive substitution of oHA for HA

**Figure 2.**
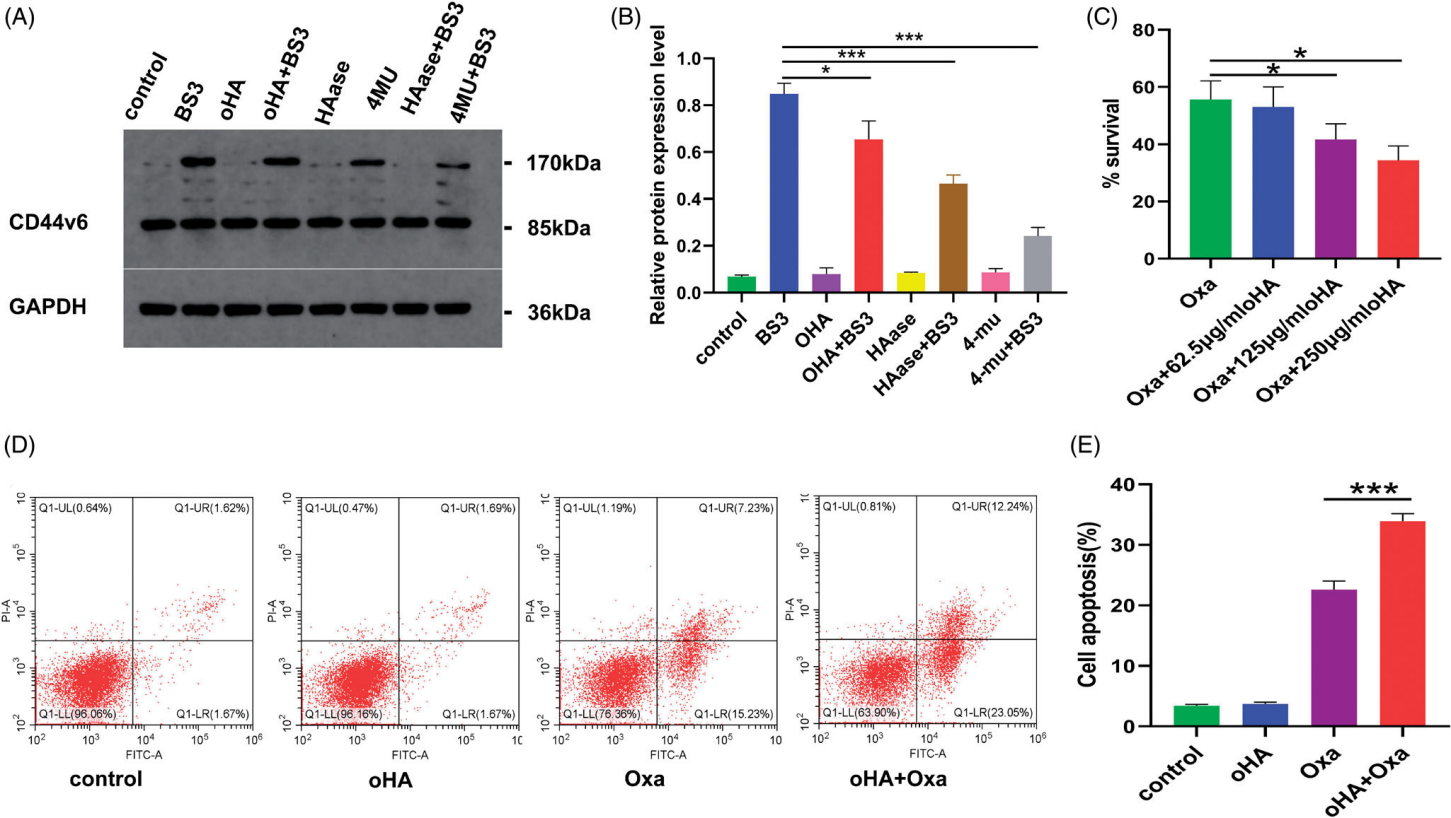
oHA competitively inhibited HA and increased the oxaliplatin sensitivity of RKO cells. (A,B) Competitive inhibition of HA by oHA in RKO cells. CD44v6 was cross-linked by BS3 treatment. When the HA synthase inhibitors 4-MU and hyaluronidase were added, the expression of CD44v6 at 170 kDa decreased, indicating that cross-linking of CD44v6 requires HA. Adding oHA, the expression of cross-linked protein is also reduced, indicating that oHA competitively inhibits HA. (C) RKO cell sensitivity to oxaliplatin in the presence versus absence of oHA treatment (*p < .05, oHA + Oxa vs. Oxa). (D-E) Flow cytometry analysis of the effect of oHA on oxaliplatin sensitivity. Oxaliplatin combined with oHA produced greater sensitivity than did oxaliplatin alone (*p < .001).

oHA is the degradation product of HA, with an effect opposite to that of HA. oHA reportedly can be used as a reverse agent for chemotherapy resistance, and smaller forms exhibit greater reversal effects (Cui et al., 2009). Therefore, the effect of oHA-4 on oxaliplatin sensitivity was investigated in this experiment. BS3 is a short-arm chemical cross-linking agent that covalently cross-links only dimer proteins naturally present on the membrane, but not two random adjacent receptor proteins. Therefore, we used western blot to detect the expression of cross-linked CD44v6 proteins at 170 kDa (Figure 2(A,B)). The expression of cross-linked CD44v6 was detected after addition of BS3 and was weakened after addition of 4-MU and hyaluronidase, indicating that CD44v6 cross-linking requires HA. Moreover, the level of this cross-linked CD44v6 protein was reduced after oHA addition, indicating that oHA competitively inhibits HA. We divided RKO cells into four treatment groups: oxaliplatin, oxaliplatin + 62.5 μg/mL oHA, oxaliplatin + 125 μg/mL oHA, and oxaliplatin + 250 μg/mL oHA. The proliferation rates of the respective groups were 56 ± 5.31%, 53 ± 5.72%, 42 ± 4.50%, and 34 ± 4.20%. Thus, when combined with oHA, oxaliplatin was more sensitive to chemotherapy (Figure 2(C)). To further clarify the effect of oHA on chemotherapy sensitivity, we divided RKO cells into the following four treatment groups: PBS, oHA, Oxa, and oHA + Oxa. We then assessed apoptosis by flow cytometry and found that the apoptosis rate was significantly higher in the oHA + Oxa group than in the Oxa group (33.91% vs. 22.60%, *p* < 0.001), implying that oHA improved oxaliplatin sensitivity (Figure 2(D,E)). oHA may reverse chemotherapy resistance for the following reasons. First, oHA is more likely to bind to CD44v6, interfering with CD44v6 cross-linking and breaking the ‘sugar coat’ barrier; this can reverse the existing CD44v6-mediated HA effect and accomplish CD44v6-mediated drug transport, greatly increasing the drug concentration in tumor cells (Toole et al., 2008). Second, oHA substantially inhibits EGFR and AKT signaling pathways in cancer stem cells and induces intracellular uptake of breast cancer resistance protein and P-gp, thus enhancing the therapeutic effects of chemotherapy drugs on malignant tumors (Gilg et al., 2008). Finally, oHA promotes the expression of p53 and PTEN, inhibits the PI3K/AKT signaling pathway, and upregulates the expression of the apoptotic protein BAD to induce apoptosis, thereby reversing chemotherapy resistance (Ghatak et al., 2002).

### 3.3. Preparation of oxaliplatin nanoparticles

In recent years, researchers have investigated safe and effective drug delivery systems to selectively kill tumor cells, which reduces the adverse effects of chemotherapy (Lim et al., 2019; Unsoy and Gunduz 2018). The ideal drug delivery system involves transport of a toxic drug to a specific target cell without leakage of the drug, followed by binding of the drug to a target cell-specific receptor, entry into the cell via internalization, and finally rapid induction of drug release via intracellular environmental stimuli such as redox potential, PH, enzymes, and temperature changes (Wei et al., 2016; Zhou et al., 2018; Razavi et al., 2020). oHA is a degradation product of HA that is widely used in drug delivery systems because it is nontoxic, non-immunogenic, and biocompatible. It also exhibits tumor-targeting capability (Chen et al., 2017; Han et al., 2019).

**Figure 3.**
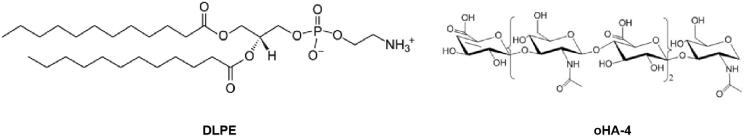
Molecular structures of DLPE and oHA-4.

In this study, oHA-4 was loaded onto the surface of oxaliplatin liposomes to prepare oHA-Lipid-Oxa nanoparticles, which enabled controlled and specific delivery and improved the sensitivity of oxaliplatin. oHA-Lipid-Oxa nanoparticles were obtained by the thin-film hydration method. Oxaliplatin nanoparticles in solution were clear and transparent. The NH_3_^+^ groups in DLPE material within oxaliplatin nanoparticles were coupled with COO- groups in oHA-4 by EDC catalysis to form oHA-modified oxaliplatin nanoparticles (Figure 3).

### 3.4. Characterization of oHA-Lipid-Oxa

**Figure 4.**
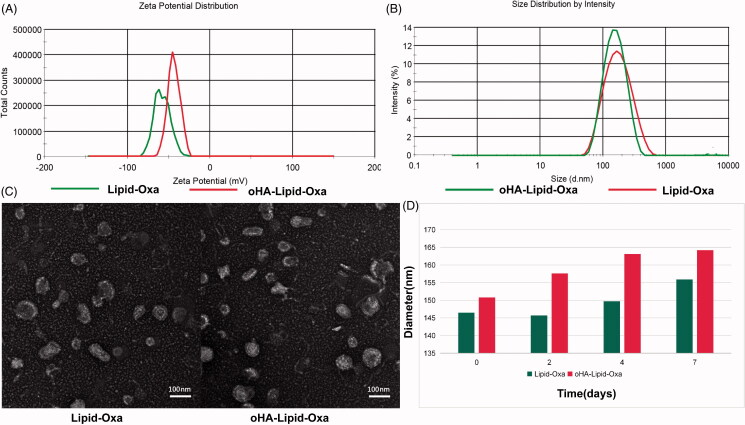
Characterization of Lipid-Oxa and oHA-Lipid-Oxa. (A) Zeta potentials of Lipid-Oxa and oHA-Lipid-Oxa. (B) Size distributions of Lipid-Oxa and oHA-Lipid-Oxa. (C) Transmission electron microscopy images of Lipid-Oxa and oHA-Lipid-Oxa. (D) Stabilities of Lipid-Oxa and oHA-Lipid-Oxa.

The particle size distribution and zeta potential of Lipid-Oxa and oHA-Lipid-Oxa were measured using a Malvern laser particle size analyzer. The respective average sizes were 146.5 ± 9.3 nm and 150.8 ± 7.2 nm (Figure 4(A)), and the respective zeta potentials were −43.6 ± 5.6 mV and −57.8 ± 7.0 mV (Figure 4(B)). The differences in liposome particle size and zeta potential indicated that oHA loading was successful. The basic characteristics of the two liposome nanoparticles are listed in Table 1.

**Table 1.** Characterization of materials.

Material	Size (nm)	Zeta (mV)	PDI	DL (%)	EE (%)
Lipid-Oxa	146.5 ± 9.3	−43.6 ± 5.6	0.202 ± 0.011	6.67	53.5
oHA-Lipid-Oxa	150.8 ± 7.2	−57.8 ± 7.0	0.233 ± 0.015	6.39	50.2

As shown in Figure 4(C), the liposome structures were observed by transmission electron microscopy. The results showed that liposome nanoparticles with spherical structures were prepared successfully and exhibited uniform morphology. Compared with unmodified oxaliplatin liposomes, the oHA-4-modified oxaliplatin liposomes had a thickened structure in the outer layer.

Oxaliplatin liposome solution was homogeneous at room temperature. Lipid-Oxa and oHA-Lipid-Oxa solutions were extracted on 0, 2, 4, and 7 day. Their particle sizes were detected using the Malvern laser particle size analyzer. The particle sizes of the Lipid-Oxa and oHA-Lipid-Oxa did not differ significantly, indicating good in vitro stability of the oxaliplatin liposomes (Figure 4(D)).

### 3.5. Determination of oxaliplatin drug loading and oHA linkage

**Figure 5.**
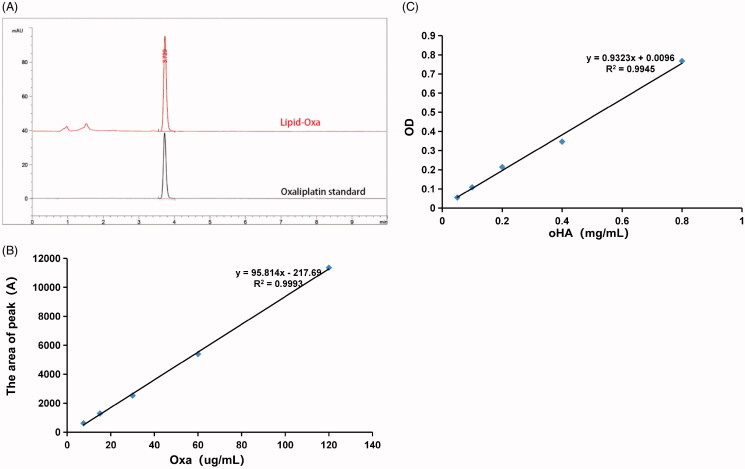
Drug loading and encapsulation efficiency of oxaliplatin in oHA-Lipid-Oxa, and the mass of oHA-4 connected. (A,B) Oxaliplatin drug loading and encapsulation efficiency detected by HPLC. (C) The mass of oHA-4 detected by carbazole sulfate.

The results of the HPLC analysis are shown in Figure 5(A). At 250 nm, the peak time of oxaliplatin standard was 3.72 mins. The absorption peak of oxaliplatin appeared at the same time, and the lipid excipients did not interfere with drug determination, which indicated that oxaliplatin was successfully encapsulated in the liposome. Moreover, a standard curve of the drug concentration (*x*) versus HPLC absorption peak area (*y*) was established using different concentrations of the oxaliplatin standard reserve solution. The standard curve was *y* = 95.814*x* − 217.69, *R*^2^ = 0.9993 (Figure 5(B)). The results showed a good linear relationship between the oxaliplatin concentration and peak area ratio in the concentration range of 7.5–120 μg/mL. The drug loading rates of oxaliplatin in Lipid-Oxa and oHA-Lipid-Oxa were 6.67% and 6.39%, respectively, and the encapsulation efficiency rates were 53.5% and 50.2%, respectively.

The concentration and absorbance of oligosaccharides were determined by ultraviolet spectrophotometry. The absorbance of oHA was detected at 490 nm, and a standard curve of the oHA-4 concentration (*x*) versus the OD value (*y*) was established. The standard curve was *y* = 0.9323*x* + 0.0096, *R*^2^ = 0.9945 (Figure 5(C)). The mass of oHA-4 in 1 mg oHA-Lipid-Oxa was 0.032 ± 0.009 mg (*n* = 3).

### 3.6. Evaluation of oHA-Lipid-Oxa *in vitro*

**Figure 6.**
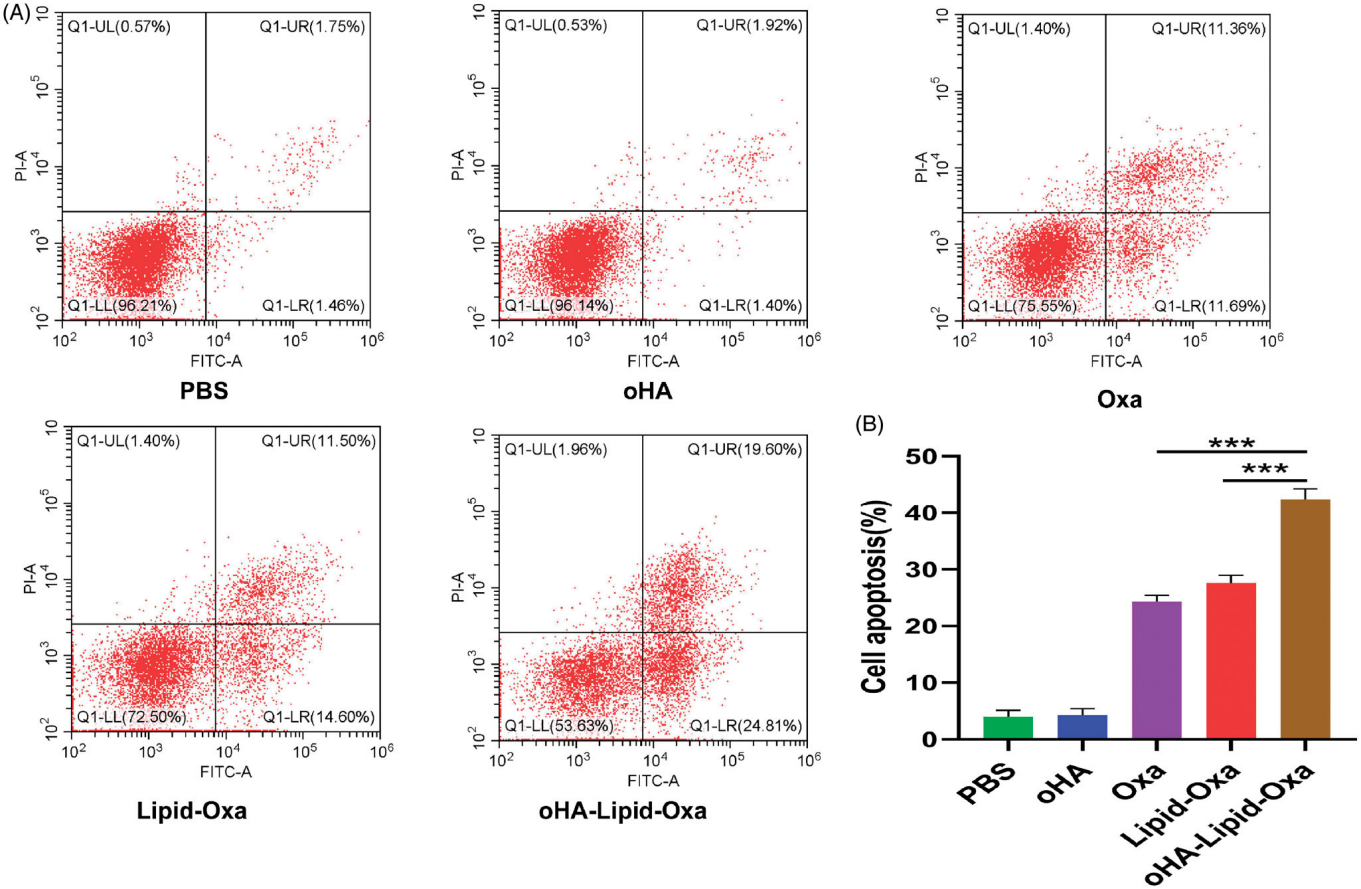
Flow cytometry analysis of the effect of oHA-Lipid-Oxa in vitro after 24 h. A. Representative flow cytometry plots. B. Ratios of apoptotic cells to total cancer cells. The anti-tumor activity in RKO cells was significantly greater for oHA-Lipid-Oxa than Lipid-Oxa or Oxa (***p < .001).

We obtained stable and homogeneous oxaliplatin nanoparticles by thin-film hydration. To evaluate the therapeutic effects of the nanoparticles, we divided RKO cells into the following five treatment groups: PBS, oHA, Oxa, Lipid-Oxa, and oHA-Lipid-Oxa. After 24 h, the apoptosis rates of the cells (determined by flow cytometry) were 3.95%, 4.25%, 24.30%, 27.60%, and 42.39%, respectively (Figure 6(A,B)). The cytotoxicity of oHA-Lipid-Oxa was higher than that of Oxa or Lipid-Oxa, potentially because oHA-Lipid-Oxa can interact with CD44v6 to improve oxaliplatin internalization in RKO cells. oHA-modified oxaliplatin nanoparticles were more effective than oxaliplatin alone in terms of cytotoxicity in vitro.

### 3.7. *In vivo* pharmacodynamics

**Figure 7.**
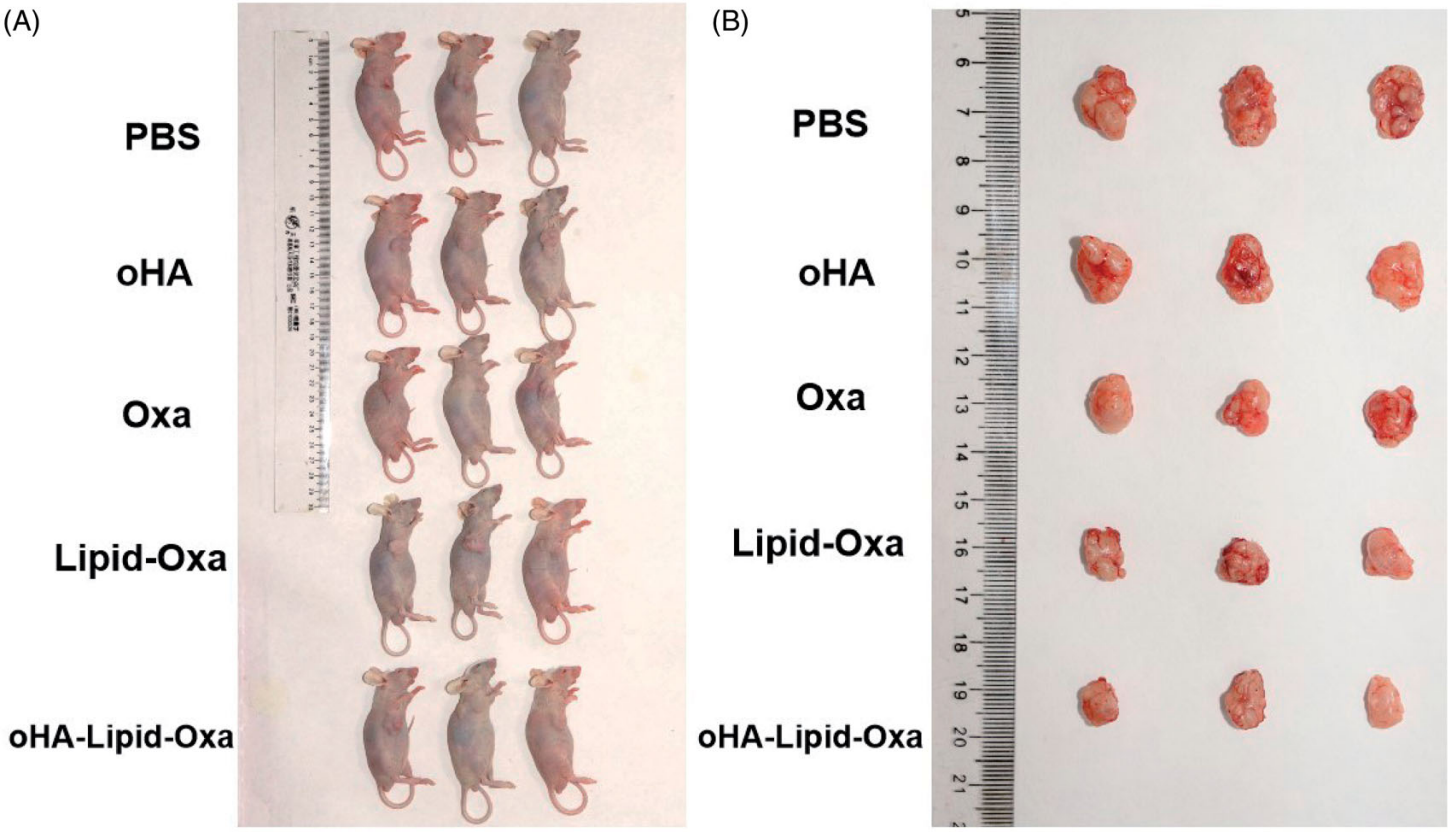
Comparison of general morphology after administration of different treatments.

**Figure 8.**
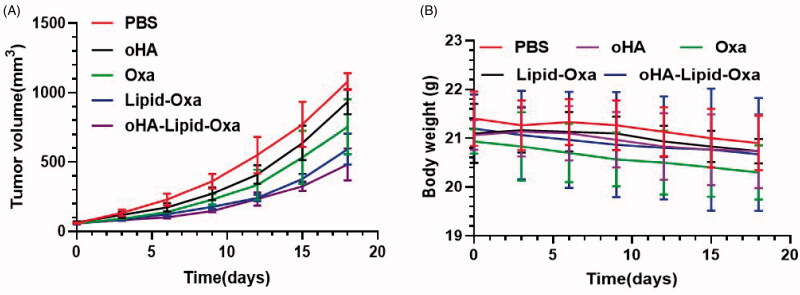
Tumor suppressive effects of Lipid-Oxa and oHA-Lipid-Oxa treatments in vivo in an RKO-bearing nude mouse model. Variations in (A) tumor volume and (B) body weight.

**Figure 9.**
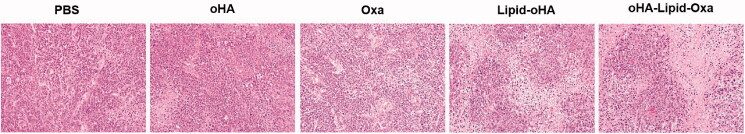
Images of hematoxylin and eosin staining (×200). (A) PBS, (B) oHA, (C) Oxa, (D) Lipid-Oxa, and (E) oHA-Lipid-Oxa.

We established a tumor-bearing nude mouse model to observe the antitumor effect of oxaliplatin nanoparticles in vivo. The tumor sizes in the nude mice of each group at the time of death are shown in Figure 7. The tumor volume was significantly reduced in nude mice treated with oHA-Lipid-Oxa compared with PBS, oHA, Oxa, or Lipid-Oxa. The tumor volumes in the PBS and oHA groups were increased significantly compared with other groups. Tumor growth was slightly inhibited in the Oxa and Lipid-Oxa groups and significantly inhibited in the oHA-Lipid-Oxa group. The anti-tumor effect of oHA-Lipid-Oxa was significantly greater than that of Lipid-Oxa (Figure 8(A)). The weight loss of nude mice in the Oxa group was substantial, but body weight did not change significantly in the oHA-Lipid-Oxa group, compared with the normal group, indicating that oHA-Lipid-Oxa had fewer side effects (Figure 8(B)). This is potentially because the activity of oxaliplatin encapsulated in liposomes is controlled, thus reducing systemic side effects. Hematoxylin and eosin staining showed that tumor cells in the PBS and oHA groups were heteromorphic, and the infiltration of lymphocytes and macrophages was more obvious in the oHA-Lipid-Oxa group than in the other groups (Figure 9). Thus, oHA-Lipid-Oxa has a greater ability to induce apoptosis and necrosis in tumor cells.

## 4. Conclusion

In this study, we found that colorectal cancer cells express higher levels of HA and CD44v6 than normal cells, which interact with each other to reduce oxaliplatin sensitivity. As an oligosaccharide fragment of HA, oHA competitively inhibits HA and improves sensitivity to oxaliplatin. In addition, oHA showed no toxicity or immunogenicity but exhibited good biocompatibility and tumor-targeting capability. Accordingly, we constructed oHA-Lipid-Oxa nanoparticles with excellent targeting and biocompatibility capabilities, which improved oxaliplatin sensitivity and suggested broad therapeutic implications.
